# Discitis due to late-onset mesh infection 14 years after inguinal hernia repair: a case report

**DOI:** 10.1186/s40792-022-01449-y

**Published:** 2022-05-25

**Authors:** Chiyo Maeda, Kai Kato, Saki Yamada, Mariko Tanaka, Ken Sujishi, Ryohei Sato, Shuichiro Takanashi, Masahiro Waseda, Tetsutaro Suzuki, Yasuo Ishida, Fumiko Kasahara

**Affiliations:** Digestive Surgery, Yokohama Asahi Chuo General Hospital, 4-20-1, Wakabadai, Asahi-ku, Yokohama, 241-0801 Japan

**Keywords:** Bacteremia, Fistula, Plug mesh

## Abstract

**Background:**

Mesh infection after inguinal hernia repair is a very rare complication. The incidence of late-onset mesh infection is approximately 0.1–0.2% of total hernia repair cases and can lead to serious complications if not treated promptly. Here, we report a rare case of discitis due to late-onset mesh infection, occurring 14 years after an inguinal hernia repair.

**Case presentation:**

An 89-year-old man was brought to our hospital with right-sided abdominal pain and signs of hypoglycemia. He had a history of type 2 diabetes mellitus and had undergone inguinal hernia repair 14 years ago. Upon admission, laboratory tests revealed no elevated inflammatory markers. Computed tomography (CT) revealed a peri-appendicular abscess. Although the patient was administered empiric antibiotics, on day 3 of admission, his white blood cell count and C-reactive protein levels increased to 38,000/µl and 28 mg/dl, respectively. CT-guided drainage was attempted but was not successful. *Escherichia coli* was detected in both blood culture collections. On day 7 of admission, the patient complained of back pain; CT on day 10 revealed a peri-appendicular abscess with a soft tissue shadow anterior to the thoracic vertebrae at the 8th/9th level. Thoracic discitis, due to bacteremia originating from the mesh abscess, was suspected. We surgically resected the appendix, followed by removal of the plug and mesh abscess. The post-operative course of the patient was uneventful. For treating discitis, it is known that antibiotic therapy is required for a minimum of 6 weeks. Therefore, on the 30th day post-surgery, the patient was transferred to the orthopedic ward for continued treatment.

**Conclusions:**

This report discusses a rare case of late-onset mesh infection leading to thoracic discitis. Since late-onset mesh infection cannot be treated solely with antibiotics, expeditious surgery should be selected when subcutaneous drainage fails. When an immunocompromised patient with bacteremia has a complaint of back pain, purulent spinal discitis should also be suspected.

## Background

Mesh hernioplasty is the most commonly performed surgery for hernia repair. The incidence of deep incisional surgical site infection (SSI) following surgical repair is approximately 0.1% to 0.2% for both open and laparoscopic hernia repairs [1]. Risk factors include: smoking, obesity, diabetes, glucocorticoid use, and a history of surgical hernia repair [2]. The optimal management for an infected mesh graft is not yet well defined in literature [3]. Here, we report a rare case of thoracic discitis in a patient with a late-onset mesh infection, occurring 14 years after an inguinal hernia repair.

## Case presentation

**Fig. 1:**
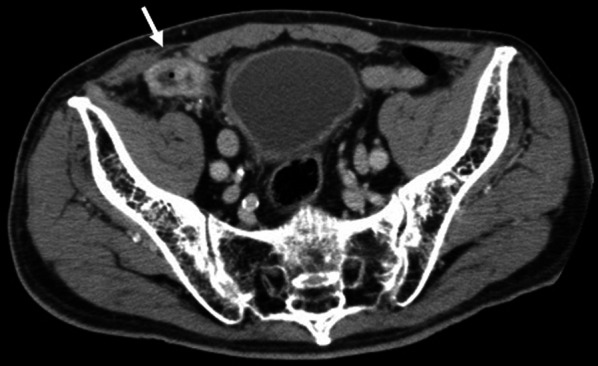
Computed tomography (CT) findings upon admission. The CT scan demonstrates a peri-appendicular abscess (arrow)

**Fig. 2:**
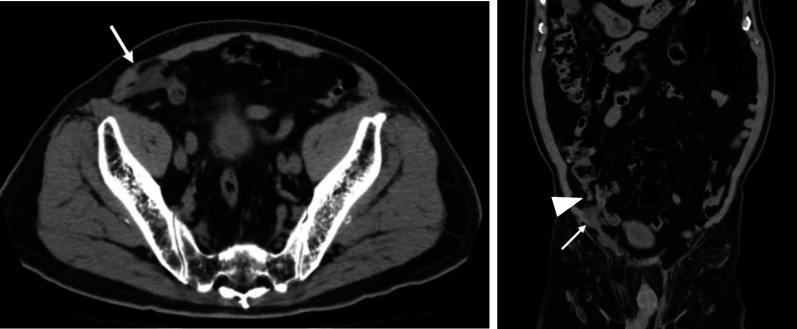
Computed tomography (CT) findings 6 years prior to surgery. **2–1** The CT scan shows fluid around the plug mesh, which was considered to be the cause of mild inflammation (arrow). **2–2** The CT scan shows that the mesh (arrow) and appendix (arrowhead) were not in contact

**Fig. 3:**
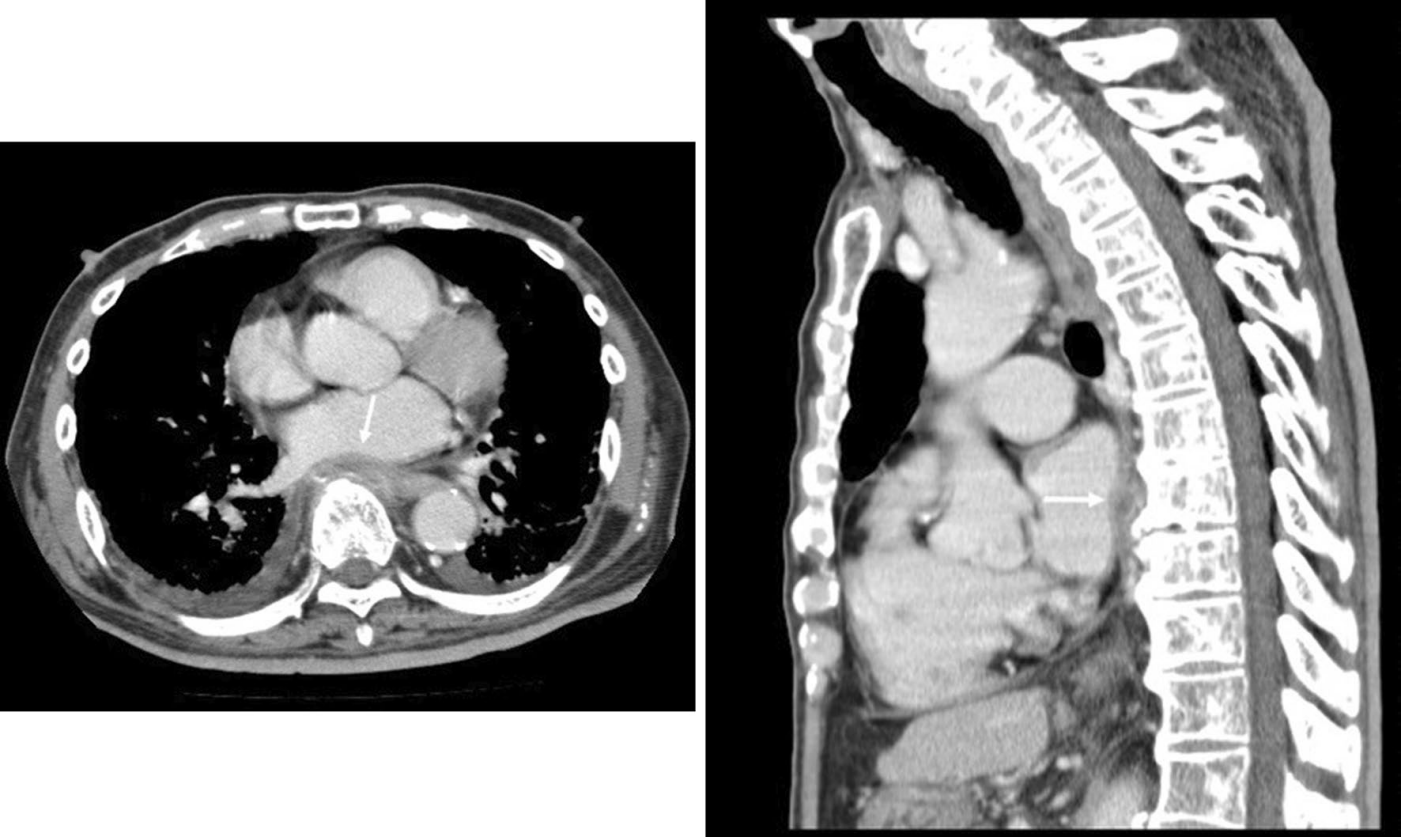
Computed tomography (CT) findings of discitis. The scan shows a soft tissue shadow in front of the vertebral body extending from the 8th/9th intervertebral space (arrows)

An 89-year-old man was brought to our hospital with right-sided abdominal pain and signs of hypoglycemia. He had a history of hypertension, interstitial pneumonia, type 2 diabetes mellitus, and dementia. He had undergone mesh plug hernioplasty for a right-sided inguinal hernia 14 years ago. On physical examination, no redness or swelling was observed in the groin region. Laboratory tests showed no evidence of elevated inflammatory markers. Initially, appendiceal abscess formation due to perforation of the appendix was suspected; however, computed tomography (CT) demonstrated a peri-appendicular abscess (Fig. 1). Thus, upon admission, we administered empiric cefalexin orally. It was impossible to visualize the abscess on ultrasound examination due to acoustic shadows caused by the inguinal mesh, indicating that the mesh and abscess were close to each other. As a result, mesh infection was suspected; however, it was difficult to distinguish between abscess formation and perforation of the appendix. Upon evaluation of the pre-operative CT, our radiologist revealed that the findings on the central portion of the appendix were suggestive of phlegmonous appendicitis and not severe appendicitis that can cause perforation. The radiologist also indicated that an abscess was formed in the plug mesh. In comparison to CT findings from 6 years ago (Fig. 2), the latest CT showed fluid collected around the mesh and an enhanced peripheral halo. Consequently, the patient was diagnosed with mesh infection, as opposed to perforation of the appendix. On day 3 of admission, his white blood cell count and C-reactive protein (CRP) levels increased to 38,000/µl, and 28 mg/dl, respectively. A full-body CT was repeated and showed no apparent source of infection except for the peri-appendiceal abscess. In addition, urinalysis did not reveal pyuria; echocardiography screening was negative for endocarditis. Hence, empiric treatment with piperacillin/tazobactam (TAZ/PIPC) was initiated, and CT-guided drainage was attempted but was unsuccessful. On the fifth day of admission, *Escherichia coli* (*E. coli*) was detected in both sets of blood cultures. Although he was taking hypoglycemic agents, including a dipeptidyl peptidase-4 inhibitor and sulfonylurea, his blood report revealed HbA1c 7.0 mmol/mol. While investigating interstitial pneumonia, partial pressure of oxygen at rest was found to be 73 mmHg at room air, and CT showed a diffuse honeycombing pattern throughout both lung fields. According to the Japanese classification, this is categorized as mild to moderate interstitial pneumonia. Therefore, general anesthesia and surgery were considered high-risk interventions in our case. Hence, we continued conservative management with antibiotics. On day 7 of admission, the patient began complaining of back pain radiating to the lower limbs; CT taken on day 10 demonstrated a peri-appendicular abscess. This was visualized as a soft tissue shadow in front of the vertebral body, extending from the 8th/9th thoracic intervertebral space (Fig. 3). Thoracic discitis, as a result of bacteremia due to the mesh abscess, was suspected. Accordingly, we decided to perform surgical drainage of the mesh abscess.

**Fig. 4:**
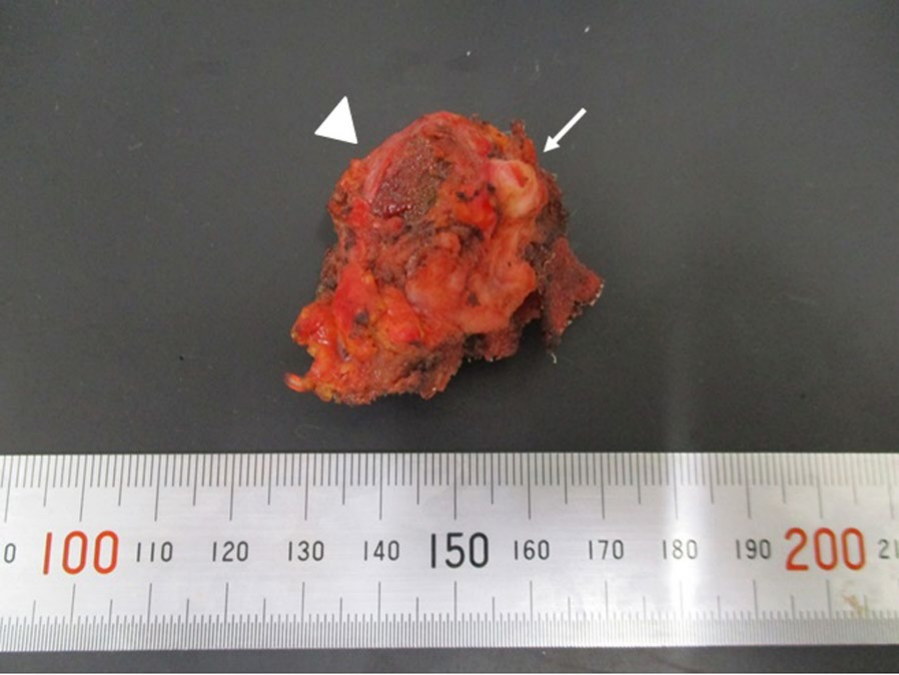
Macroscopic findings of the abscess. Macroscopic examination shows a single mass containing the underlay mesh (arrowhead) and appendix (arrow)

**Fig. 5:**
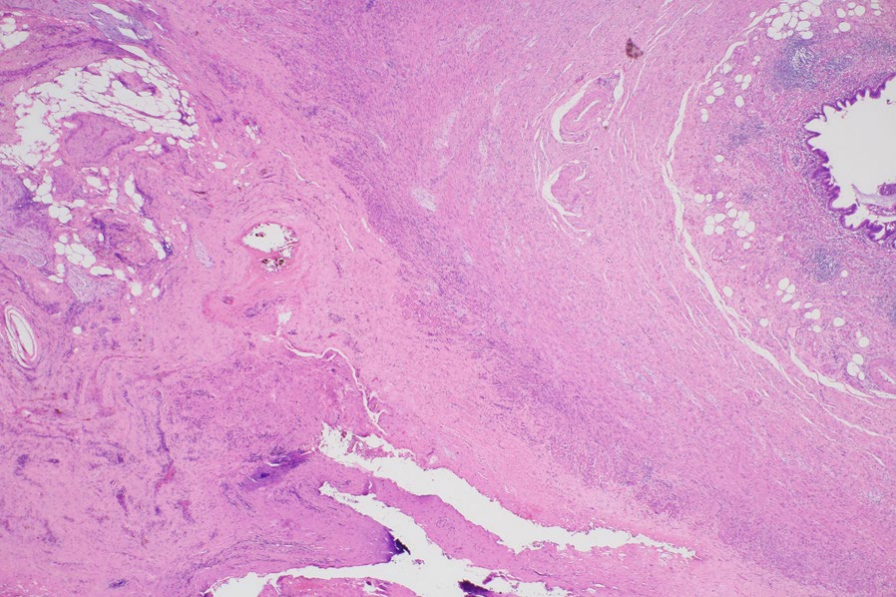
Histological findings of the appendix. The appendiceal mucosa is relatively normal, and the serosal side shows thickened fibrotic changes. Hemosiderin deposition is seen on the mesenteric surface, suggesting chronic inflammatory changes. There are no signs of perforation

Laparotomy with a midline incision was performed under general anesthesia. A single mass was observed, containing the underlay mesh (plug) and the appendix. Pus oozed out, and a sample was obtained for bacterial culture. The plug mesh encompassed the appendix; thus, removal of the plug and an appendectomy were indicated. An on-lay mesh was tightly adhered to the internal oblique muscle. The operative time was 78 min, and the volume of blood loss was 10 ml. The pus culture revealed growth of *Citrobacter freundii* and *Bacteroides caccae*, both indigenous bacteria found in the human intestinal tract. Macroscopic examination showed the plug mesh and appendix to have formed into a single mass (Fig. 4). Histologically, the appendiceal mucosa was relatively normal; however, thickened fibrotic changes were observed on the serosal side (Fig. 5). Furthermore, hemosiderin was deposited on the mesenteric surface, suggesting chronic inflammatory changes within the abdominal cavity. These findings suggest that the patient suffered from chronic secondary appendicitis accompanied with fibrotic thickening and peritonitis. Blood culture taken on the 13^th^ day after surgery was negative for bacteremia. TAZ/PIPC was administered for 9 days, followed by sulbactam sodium/ampicillin sodium (SBT/ABPC) for 38 days. Later, minocycline was also incorporated into the treatment plan. The complication of discitis indicates a long-term course of antibiotics, requiring at least 6 weeks. It is to be noted that the inguinal hernia had not recurred, and the patient was transferred to the orthopedic ward on the 30^th^ day for continued treatment. On day 50 post-surgery, the patient’s back pain was alleviated; however, CT performed on day 60 showed the findings of discitis to be unchanged. Eventually, a repeat CT taken on day 79 showed some resolution of discitis. Unfortunately, our patient died on the 80th day post-surgery due to aspiration pneumonia.

## Discussion

The definition of late-onset mesh infection is not clearly stated in the literature, but the Center for Disease Control and Prevention guidelines state that SSI occurs within 90 days of herniorrhaphy [4]. Therefore, infection presenting 4 months after herniorrhaphy is considered a late-onset mesh infection.

There are two hypotheses for abscess formation between the mesh and appendix. The first hypothesis proposes fistula formation due to chronic friction between the mesh and appendix. A plug mesh protrudes into the abdominal cavity and is more likely to cause friction than any other mesh. This appears to result from erosion of the plug due to its conical shape and heavy weight [5]. A CT scan taken 6 years before the surgery (Fig. 2 2-1) showed fluid around the plug mesh, which implied inflammation. In addition, at that time, the mesh and the appendix were not in contact (Fig. 2 2-2). Of the 47 cases of late-onset mesh infection reported by Jin et al. [5], 4 cases revealed a fistula between the mesh and intestine during surgery. However, the fistula could not be identified during surgery, since the appendix and mesh were tightly adhered. In the same study by Jin et al., 46 of 47 (97.9%) showed local inguinal skin swelling and erythema [5]. In our case, there was no local swelling or redness. The second hypothesis postulates the spread of inflammation due to appendicitis itself or perforation of the appendix. The inflammatory response rose rapidly, and it is possible that the appendix had perforated and formed an abscess around the mesh. Negative findings to this hypothesis were that CT and pathological results showed no strong inflammation of the mucosal side of the appendix. However, inflammation on the serosal side was seen in the pathology. From the above, it was difficult to select one acceptable hypothesis.

Here, *E. coli* was detected in the blood culture; *Citrobacter freundii* and *Bacteroides caccae* were found in the abscess culture. Since infection originated from the appendix, a mixed etiology with multiple bacterial species was confirmed. Microbial substitution as a result of antibiotics was thought to be the reason for the detection of different organisms in the blood and abscess culture. Typically, bacteria involved in mesh infections are those with the ability to form biofilms, such as coagulase-negative staphylococci and *Staphylococcus aureus *(*S. aureus*) [6]. Enteric gram-negative bacteria and anaerobes are also potential pathogens [6]. However, the presence of a pathogen does not indicate the need for mesh excision [6]. The optimal management of an infected mesh graft is not well defined [3]; however, patients with suspected mesh infection should initially be started on empiric broad-spectrum parenteral antibiotics, such as SBT/ABPC or TAZ/PIPC. Following this, the results of fluid or tissue culture and antibiotic sensitivity testing should guide the subsequent choice of therapy [6].

Of the previously reported cases of late-onset mesh infection, surgical removal was performed in all cases [7]. On the other hand, a retrospective study evaluated the outcomes in 21 patients who underwent percutaneous drainage for mesh-related fluid collections. Of the 21, 12 (57%) patients were treated successfully with percutaneous drainage and antibiotics [8], suggesting that surgical removal is not always necessary. However, late-onset mesh infection cannot be treated with antibiotics alone, and mesh removal surgery is indicated upon failure of CT-guided drainage [5]. Moreover, patients presenting with systemic signs of sepsis and local evidence of infection should undergo immediate surgical drainage and mesh removal [9]. In our patient, the development of discitis could have been avoided if the abscess had been surgically drained when elevated CRP levels were noted. Therefore, surgery must be considered in patients with pre-operative complications and the elderly.

Although mesh removal can potentiate hernia recurrence, Yang et al. [10] reported that merely 29 (7.4%) of the 392 cases of mesh removal had a recurrence of inguinal hernia. In our case, the plug mesh was completely removed, and part of the on-lay mesh was retained due to strong adhesions with the tissue.

Discitis is an infection of the intervertebral disc space. Discitis and vertebral osteomyelitis can occur together or independently. The term “pyogenic spondylitis” refers to both vertebral osteomyelitis and discitis. In most cases, the diagnosis and management of both conditions are similar [11]. Spontaneous discitis usually occurs due to hematologic spread, most commonly from a urinary or respiratory infection [11]. The major clinical manifestation of discitis is neck or back pain, with or without fever. Therefore, when an immunocompromised patient with bacteremia has a complaint of back pain, purulent spinal discitis should also be suspected. The most common laboratory abnormalities include an elevated erythrocyte sedimentation rate and CRP level. Although magnetic resonance imaging (MRI) is the most sensitive radiographic technique for the diagnosis of discitis [12], our patient had dementia; thus, MRI could not be performed. Typical findings on contrast-enhanced CT in discitis and vertebral osteomyelitis include a soft tissue shadow and fluid collection anterior to the thoracic spine [13].

The most common causative organism of vertebral osteomyelitis is *S. aureus,* accounting for more than 50% of cases in most developed countries [11]. Other causes include enteric Gram-negative bacilli, non-pyogenic *Streptococci,* and pyogenic *streptococci*. If blood culture is positive for a likely pathogen (such as *S. aureus*), a needle biopsy may not be necessary for patients with suspected osteomyelitis [14]. Discitis is routinely treated with antibiotics for a minimum of 6 weeks, and a careful review is undertaken to determine whether further treatment is required [10, 14]. Discitis is associated with severe pain that hampers routine activities of the patient; therefore, prompt elimination of the source of bacteremia is essential to alleviate symptoms.

## Conclusions

This report herein discussed a rare case of late-onset mesh infection leading to thoracic discitis. Since late-onset mesh infection cannot always be treated solely with antibiotics, expeditious surgery should be indicated when subcutaneous drainage fails. When an immunocompromised patient with bacteremia has a complaint of back pain, purulent spinal discitis should also be suspected.

## Data Availability

Not applicable.

## Abbreviations

SSI: Surgical site infection
CT: Computed tomography
CRP: C-reactive protein
TAZ/PIPC: Tazobactam/piperacillin
SBT/ABPC: Sulbactam sodium/ampicillin sodium
*E. coli*: *Escherichia coli*
*S. aureus*: *Staphylococcus aureus*
MRI: Magnetic resonance imaging
